# The Healthy Primary School of the Future: study protocol of a quasi-experimental study

**DOI:** 10.1186/s12889-016-3301-9

**Published:** 2016-07-26

**Authors:** M. Willeboordse, M. W. Jansen, S. N. van den Heijkant, A. Simons, B. Winkens, R.H.M. de Groot, N. Bartelink, S. P. Kremers, P. van Assema, H. H. Savelberg, E. de Neubourg, L. Borghans, T. Schils, K. M. Coppens, R. Dietvorst, R. ten Hoopen, F. Coomans, S. Klosse, M.H.J. Conjaerts, M. Oosterhoff, M. A. Joore, I. Ferreira, P. Muris, H. Bosma, H. L. Toppenberg, C. P. van Schayck

**Affiliations:** 1 Department of Family Medicine, School of Public Health and Primary Care (CAPHRI), Maastricht University, Maastricht, The Netherlands; 2 Academic Collaborative Centre for Public Health Limburg, Public Health Services, Geleen, The Netherlands; 3 Department of Health Services Research, CAPHRI, Maastricht University, Maastricht, The Netherlands; 4 MOVARE Educational board, Kerkrade, The Netherlands; 5 Department of Methodology and Statistics, CAPHRI, Maastricht University, Maastricht, The Netherlands; 6 Welten Institute - Research Centre for Learning, Teaching and Technology, Open University of the Netherlands, Heerlen, The Netherlands; 7 School of Nutrition and Translational Research in Metabolism (NUTRIM), Maastricht University, Maastricht, The Netherlands; 8 Department of Health Promotion, CAPHRI, Maastricht University, Maastricht, The Netherlands; 9 Department of Health Promotion, NUTRIM, Maastricht University, Maastricht, The Netherlands; 10 Department of Human Movement Sciences, NUTRIM, Maastricht University, Maastricht, The Netherlands; 11 School of Business and Economics, Maastricht University, Maastricht, The Netherlands; 12 Department of Law, Maastricht University, Maastricht, The Netherlands; 13 Academic Hospital Maastricht, Treatment and Care Unit, Maastricht, The Netherlands; 14 Department of Clinical Epidemiology and Medical Technology Assessment, CAPHRI, Maastricht University, Maastricht, The Netherlands; 15 School of Public Health, The University of Queensland, Herston, Brisbane Australia; 16 Department of Clinical Psychological Science, Faculty of Psychology and Neuroscience, Maastricht, The Netherlands; 17 Department of Social Medicine, CAPHRI, Maastricht University, Maastricht, The Netherlands

**Keywords:** Academic Achievement, Accelerometer, Children, Primary school Intervention, Nutrition, Obesity, Physical activity, Prevention, School health

## Abstract

**Background:**

Unhealthy lifestyles in early childhood are a major global health challenge. These lifestyles often persist from generation to generation and contribute to a vicious cycle of health-related and social problems. This design article presents a study evaluating the effects of two novel healthy school interventions. The main outcome measure will be changes in children’s body mass index (BMI). In addition, lifestyle behaviours, academic achievement, child well-being, socio-economic differences, and societal costs will be examined.

**Methods:**

In close collaboration with various stakeholders, a quasi-experimental study was developed, for which children of four intervention schools (*n* = 1200) in the southern part of the Netherlands are compared with children of four control schools (*n* = 1200) in the same region. The interventions started in November 2015. In two of the four intervention schools, a whole-school approach named ‘The Healthy Primary School of the Future’, is implemented with the aim of improving physical activity and dietary behaviour. For this intervention, pupils are offered an extended curriculum, including a healthy lunch, more physical exercises, and social and educational activities, next to the regular school curriculum. In the two other intervention schools, a physical-activity school approach called ‘The Physical Activity School’, is implemented, which is essentially similar to the other intervention, except that no lunch is provided. The interventions proceed during a period of 4 years. Apart from the effectiveness of both interventions, the process, the cost-effectiveness, and the expected legal implications are studied. Data collection is conducted within the school system. The baseline measurements started in September 2015 and yearly follow-up measurements are taking place until 2019.

**Discussion:**

A whole-school approach is a new concept in the Netherlands. Due to its innovative, multifaceted nature and sound scientific foundation, these integrated programmes have the potential to form a template for primary schools worldwide. The effects of this approach may extend further than the outcomes associated with well-being and academic achievement, potentially impacting legal and cultural aspects in our society.

**Trial registration:**

The study protocol was registered in the database ClinicalTrials.gov on 14-06-2016 with the reference number NCT02800616.

## Background

Unhealthy lifestyles, bred by low levels of physical activity and unhealthy dietary behaviours, are a persistent problem in Western societies [1]. Such lifestyles are usually adopted at a young age, and often persevere into adulthood [2]. Even in early childhood, unhealthy behaviours contribute to major issues such as a reduced health-related quality of life (HR-QoL), psychosocial problems, and chronic diseases including obesity [3–5]. In the long run, this often results in higher health care costs, reduced academic achievement, reduced labour participation, and a lower socio-economic status (SES) [5, 6]. A vicious circle is likely to emerge, transferring problems from one generation to the next. It is clearly a major challenge for public health professionals to break this tenacious pattern.

In efforts to reverse the public health epidemic, the school setting is an ideal environment for promoting healthy lifestyles. A school setting enables multiple aspects of health promotion (education, social environment, physical environment, school health policy, parenting) to be modified, thereby facilitating successful implementation [7]. For instance, the school setting offers the advantages of facilitating compliance with an intervention and also enables preventive measures to reach children from a variety of socio-economic and ethnic backgrounds [8, 9]. The Dutch primary school system in particular, leaves much room for improvement with respect to nutrition, and physical activity during the school day [10]. In addition, there is some preliminary evidence that lifestyle interventions improve academic achievements, thereby helping schools to achieve their primary academic goals [11–13].

Worldwide, many school-based health interventions have been evaluated [14]. Over 80 % of these interventions reported at least one positive effect on risk factors for non-communicable diseases (e.g. obesity, physical inactivity, smoking, inadequate or poor nutrition) [15]. Despite the relatively high costs of school-based interventions, existing programmes have been found to be cost-effective in terms of body mass index (BMI) improvements and HR-QoL gains [16, 17]. Normalized BMI distributions, gathered across the entire school population, provide an informative outcome measure for the evaluation of such interventions. Newly learned healthy lifestyle habits are likely to lead to favourable weight changes among overweight and obese children, as well as underweight children, but are also likely to stimulate children with a healthy weight to maintain their weight. Admittedly, the potential of school-based interventions to normalize BMI is found to be modest [14, 15, 18]. The most frequently reported reasons for this modest effectiveness are low parental involvement, a short duration of the intervention, and a lack of a whole-school approach [14, 15, 18–21]. It is often considered difficult to acquire the necessary support and deal with the high costs and efforts that need to be made to implement multicomponent interventions. As a result, schools often choose less intense single-component interventions. Physical education seems to be one of the most viable components to effectively decrease obesity rates in schools [20, 22]. Despite the implementation benefits of single-component interventions, however, a whole-school approach should always be preferred, as newly acquired lifestyle habits are more likely to last if embedded within an integrated approach.

We present a study protocol that examines the effectiveness of two novel, integrated healthy school interventions. One is a full intervention called ‘The Healthy Primary School of the Future’, the other is a partial intervention called ‘The Physical Activity School’. These intervention approaches will be compared with the regular school approach that is currently common practice in the Netherlands. We hypothesize that these healthy school interventions will result in normalized BMI distributions that are more in line with national and international standards (smaller standard deviations) among primary school children, with a more pronounced effect in the full intervention schools (due to the expected synergy between exercise and diet) than in the partial intervention schools. Also, our multi-disciplinary research group will study a wide range of outcome measures, including lifestyle behaviours, academic achievement, child well-being, socio-economic differences, and societal costs. Moreover, an evaluation will be performed of the legal consequences of a healthy school approach in the Netherlands, as well as the conflicting interests of the stakeholders.

Our primary research question is: What is the effect of the full intervention (‘The Healthy Primary School of the Future’) on the BMI of primary school children compared to no intervention (control schools)? Our secondary research question is: What is the effect of the full intervention on the BMI of primary school children compared to the partial intervention (‘The Physical Activity School’)?

Our tertiary research questions are: (1) What is the effect of the full intervention in comparison with the partial intervention and the regular school approach (control schools) on: (a) children’s levels of physical activity and sedentary behaviour, nutritional knowledge, healthy food preferences and behaviour, cognitive and non-cognitive performance, HR-QoL, socio-emotional development, and sick leave? (b) parenting and teacher practices regarding physical activity and nutrition? (c) parental HR-QoL, well-being, labour participation and sick leave? (d) benefits across different socio-economic backgrounds? (e) long and short term cost-effectiveness? (f) satisfaction among the involved stakeholders (children, parents, teachers, and child care partners)? (2) Which determinants influence the quality of the implementation of the intervention? (3) What is the scope of children’s human rights to health, what is the legal role of primary schools in realizing these rights (e.g., obligations and responsibilities of state and non-state actors, conflicts of interests and legal solutions to these conflicts), and is the intervention feasible within Dutch educational law?

## Methods

### Setting and study design

The current study has a quasi-experimental design with four intervention schools and four control schools. All participating schools are primary schools situated in the Parkstad region in the Province of Limburg, in the southern part of the Netherlands. This is a relatively poor region with a low SES in which unhealthy lifestyles are highly prevalent [23, 24]. Overweight and obesity rates are substantially higher than the national average in the Netherlands [25]. The intervention schools and control schools are both members of the MOVARE educational board. In the Dutch primary school structure, pupils successively follow eight ‘groups’, starting in group 1 at the age of 4 and typically proceed to secondary school at the age of 11 or 12. Internationally, the first two groups are comparable to pre-school and the last six groups are comparable to grades 1–6.

**Fig. 1:**
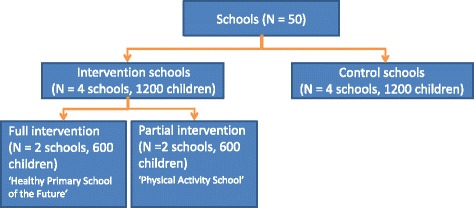
Study design

Two schools take part in the full intervention named ‘The Healthy Primary School of the Future’. Two other schools implement the partial intervention named ‘The Physical Activity School’ (Fig. 1). No randomization was applied, because full voluntary cooperation and participation of the intervention schools is required for the implementation of the interventions. The population consists of a dynamic cohort comprising children in groups 1–8 (Table 1). Baseline measurements were conducted in September-October 2015, and yearly follow-up measurements will take place until 2019. The intervention started in November 2015.

**Table 1:** Dynamic study cohort

Cohort	T1 2015/2016	T2 2016/2017	T3 2017/2018	T4 2018/2019	T5 2019/2020	Exposure in years
A					Group 1	0
B				Group 1	Group 2	1
C			Group 1	Group 2	Group 3	2
D		Group 1	Group 2	Group 3	Group 4	3
E	Group 1	Group 2	Group 3	Group 4	Group 5	4
F	Group 2	Group 3	Group 4	Group 5	Group 6	4
G	Group 3	Group 4	Group 5	Group 6	Group 7	4
H	Group 4	Group 5	Group 6	Group 7	Group 8	4
I	Group 5	Group 6	Group 7	Group 8	Group 8 ^a^	4
J	Group 6	Group 7	Group 8			3
K	Group 7	Group 8				2
L	Group 8					1

^a^In cohort I, T5 measurements will be conducted in June 2019 instead of September -November 2020

Almost all schools in the southern part of the Netherlands participate in the health monitoring programme of the regional Public Health Services (GGD) and the OnderwijsMonitor Limburg, a collaboration between Maastricht University, schools and school boards to collect data from children at school in Limburg [26, 27]. This allows us to put the measures of this intervention into the perspective of the total school population of the wider Limburg region.

### Recruitment of schools and subjects

#### Recruitment of intervention schools

In March 2013, 12 out of 53 schools governed by the MOVARE educational board were informed about the study. These schools were selected because of their supportive geographical environment (e.g., sufficient sports facilities in the area and supportive local politicians). Out of these 12, we aimed to recruit four large intervention schools with a minimum of 140 children per school in groups 2, 3, 4 and 5. Four schools gave their initial consent and started to raise support from all stakeholders (e.g., parents, children, partners of the school) and to develop the intervention together with partners. Eventually, two of the four schools gave their approval for the full intervention. A third school received enough support from stakeholders to implement the partial intervention and a fourth school dropped out because of a lack of support among school staff and parents. After the withdrawal of this school, another school from the same educational board was recruited to implement the partial intervention. The recruitment of all intervention schools was completed in July 2015.

#### Recruitment of control schools

From all schools of the MOVARE educational board, four control schools were recruited with a minimum of 140 children per school in groups 2, 3, 4 and 5. All schools that voluntarily agreed to participate could join the study, and no inclusion or exclusion criteria were set. The recruitment of the control schools was completed in January 2015. The control schools are characterized by a school environment that is representative of schools in the Netherlands in terms of lifestyle education, school hours and number of physical education (PE) classes.

The intervention and control schools are comparable in terms of socioeconomic status and there are no statistically significant differences in academic performance, as measured at baseline by the so-called CITO scores (see the section on Children's academic achievements).

#### Recruitment of subjects

The recruitment of the children for the baseline measurement was completed in October 2015. Until 2019, all new children entering the intervention and control schools will be invited to participate in the study (Table 1). For the baseline measurement, children were recruited by means of information brochures sent by mail, and reminders by the school staff. In addition, the research team visited all classrooms to inform children about the study and to encourage them to participate. All children of the intervention and control schools are eligible to participate in this study (Table 1). Children who switch schools during the four-year study period will not be followed up with the extensive measurement programme, but we do have the outcome variables collected by the OnderwijsMonitor Limburg and GGD for these children.

### Interventions

The interventions are based on the socio-ecological approach to school health promotion, focusing on the wider picture of interrelationships between individuals with their personal characteristics (e.g., children and their parents), and their environment (e.g., the school and its neighbourhood) [7]. The interventions focus on multiple aspects (education, social environment, physical environment, school health policy, parental practices) and aim to influence multiple settings (school, home environment, school neighbourhood).

Both school interventions, ‘The Healthy Primary School of the Future’ and ‘The Physical Activity School’, involve making changes to school hours and physical activity. School hours are extended to facilitate implementation of the interventions. Children attend school from 08:30 to approximately 15:30/16:00 instead of 8:30 to 12:30 and 13:30 to 15:00. These school hours allow educational activities to be more in line with children’s bio-rhythm [28]. Where possible, schools use the 10:00–12:00 and 14:00–15:30/16:00 time-slots for cognitive tasks, as previous studies suggest that children can concentrate better during these timeframes than during those used in regular school environments [28]. With regard to physical activity, the schools offer a structured programme including sports, play and creativity for at least 1 hour a day for 4 days a week, guided by the pedagogical staff (Table 2).

**Table 2:** Compulsory changes and possible additional activities to promote a healthy lifestyle

Theme	Situation of control schools	Compulsory changes	HPSotF	PAS	Additional activities in HPSotF and PAS
School hours	➢ 08:30–14:30/15:00	➢ 08:30–15:30/16:00	X	X	
	Obligatory lessons. Lunch break for lunch from school 30–60 min.	Obligatory lessons. Lunch break and organised sports, free play and cultural activities of 75–90 min.			
School health policy	➢ No school health policy.				Changing existing policy into healthy lifestyle stimulating policy.
Healthy lifestyle education	➢ No nutritional and/or physical activity education.				➢ ‘Lekker Fit’ program: healthy lifestyle education with physical activity and nutrition lessons as part of the curriculum [60].
Physical activity	➢ 30-min lunch break with free play.	➢ Organised sport, free play and cultural activities of at least 60 min a day.	X	X	➢ ‘By Foot And By Bike’ program: between-class competition focusing on active transport.
	➢ 1 h PE/week.				➢ Energizers: introducing short breaks of physical activity during lessons.
	➢ No physical activity breaks.				➢ Increased intensity of PE lessons [61].
	➢ School yard with limited physical activity stimulating facilities.				➢ At least 2 PE lessons a week
					➢ Introducing swimming lessons.
					➢ Schoolyard with a physical activity stimulating environment.
					➢ Introduction lessons by sports clubs.
Nutrition	➢ Children eat foods brought along from home at school or they have lunch at home.	➢ Healthy lunch and morning snack provided by school.	X	-	➢ ‘Smaaklessen’ program: practical food tasting lessons [62].
					➢ Keeping a vegetable garden at the school yard.
					➢ Distribution of water bottles.
Socio-emotional well-being	-				➢ ‘Taakspel’ program: a group-based approach in which children learn to comply better with rules in the classroom [63].
Parents	-				➢ Website with general advice for healthy practices at home.
					➢ ‘Gezonde afspraken met je kind’: e-health program to make healthy agreements with one’s child.
					➢ ‘Goedkoop Gezonde Voeding’ program: learning about cheap and healthy nutrition.
					➢ Interactive theatre: interactive evening to discuss (obstacles of) healthy practices at home.
					➢ ‘COOL’ program: lifestyle intervention for overweight children and their parents
					➢ ‘Lifestyle Triple P’ program: lifestyle intervention for parents of overweight children.

*Abbreviations*: *HPSotF* healthy primary school of the future, *PAS* physical activity school, *PE* physical education

The full intervention schools provide a healthy morning snack and healthy lunch every day. Lunch is provided in the classroom or in a central location in the school. A variety of food products from all food groups are available, from which children can choose. The products are carefully selected to meet the guidelines of the Netherlands Nutrition Centre Foundation. Pedagogical staff will supervise the lunch sessions to make sure that the children choose products from all recommended food groups and that they choose the recommended portion sizes. The lunch cycle changes every 10 weeks.

In addition to these compulsory implementations, both the full and the partial intervention schools can choose to offer additional activities (Table 2). To support and encourage the schools, we searched for relevant and evidence-based additional activities, using a systematic health promotion approach, based on the PRECEDE-PROCEED model [7, 29] and the Intervention Mapping protocol [30]. A diagnosis of the health problems (step 1) and associated behavioural and environmental factors (step 2) resulted in behaviour goals, e.g. children using active transport to school and children eating two pieces of fruit a day. We also searched the literature for determinants that influence these behaviour goals (step 3). Next, the resulting overview of determinants was used to search for evidence-based activities or best practices available nationally or internationally (step 4) [31]. Schools are free to choose the activities which best fit their possibilities and goals. Employees of the regional Public Health Services (GGD) and community sports coaches support stimulate the school in this decision-making process.

Additional activities focus on school health policy. Creating policy on health topics, for example on healthy birthday treats, should clarify what social norms are important to the school, making the communication with parents easier and more consistent. Additional activities also focus on health education. A healthy lifestyle education programme is expected to promote healthy nutrition (fruit, vegetables, fibre-rich products, lower fat and lower sugar products and normalized portion sizes), physical activity (reduction of sedentary behaviour, increase in moderate- to high-intensity physical activities, compliance with the Dutch standards for healthy behaviour), and socio-emotional well-being (increased self-esteem, executive control function, decreased bullying). The educational activities incorporate interactive lessons, and games to increase knowledge of, and familiarity with, healthy food products and physical activities. Schools intend to set aside at least 1 hour per week in the school curriculum for health education.

Additional activities are implemented with the aim of creating an optimal social and physical environment to make healthy choices easier. For instance, schools can choose to increase PE classes and/or increase the intensity of the existing PE classes. The schoolyard can be equipped with low-cost facilities to encourage physical activity, such as play equipment and balls. Sports clubs can be introduced to the schools to give introductory lessons to the children. Short physical activity breaks can be incorporated during lessons in order to reduce sedentary time. These breaks can occur in the classroom (e.g. games involving physical movements, interactive dances), or outside in the schoolyard. Regarding nutrition, the schools are stimulated to choose additional activities such as food tasting, gardening (i.e., growing vegetables), and the distribution of water bottles.

Finally, several additional activities are aimed at parents to stimulate parental involvement and encourage the successful transfer of a healthy lifestyle from school to home.

### Outcome measures

An overview of all outcome measures and the corresponding informants (e.g., child, parent, teacher, or other) is presented in Table 3. All measurements will be completed yearly unless stated otherwise. Measurements involving the children will be obtained in groups, during regular classes, except for the anthropometric measurements. All questions will be tailored to the different age categories and include numerous graphical illustrations. No open-ended questions will be used. All questionnaires have been pretested several times in comparable populations and settings. In the youngest groups (groups 2–4), the questions will be read out by the teacher or a research assistant, and answered in writing by the children. The oldest children (groups 5–8) will read and fill in the questionnaires themselves. During the completion of the questionnaires, a teacher and at least one researcher will be present to monitor the process and to answer questions. The majority of the child-related measurements will be scheduled during one regular school week to avoid overburdening the children and school staff. Children in group 1 will be excluded from most measurements, as their young age (4 years in September) complicates most of the measurements. Also, not all group 1 children will attend school yet during the measurement periods (September-October), as children in the Netherlands gradually enter group 1 during the school year, depending on their birthday. Parents of children in groups 1–8 will receive an online questionnaire and teachers of all classes will receive a short questionnaire on paper. Privacy-sensitive questions, such as parental BMI, SES and disease status will not be mandatory to complete.

**Table 3:** Overview of all the outcome measurements and their corresponding sources

Outcome measurement	Informants					
	Children			Parents of children in groups 1–8 (age 4–12)	Onderwijs monitor Limburg	GGD
	Group 2 (age 5–6)	Group 3–4 (age 6–8)	Group 5–8 (age 8–12)			
General health child						
Weight, objectively measured	X	X	X			
Height, objectively measured	X	X	X			
Waist and hip circumference, objectively measured	X	X	X			
Handgrip strength, objectively measured	X	X	X			
Blood pressure						X
Disease status, hospital admissions, medicine use, healthcare visits				X		X
Sick leave					X	
Birthweight						X
Socio-emotional health of child						
HR-QoL (EQ-5D-Y, PedsQL)			X	X		
Psychological attributes (SDQ)			X	X		
Self-efficacy (SEQ-C and Manikin scale, full SEQ-C only in groups 7–8)			X			
Self-confidence, social skills, school wellbeing, future expectations and social support					X	
Physical activity behaviour of child						
Physical activity (Actigraph)		X	X			
Sedentary behaviour (ActivPal)			group 5			
Shuttle run test		X	X			
Sports club membership		X	X	X		
Active transport forms to school	X	X	X			
Leisure time physical activity	X	X	X			
Leisure time physical activity				X		
Dietary behaviour of child						
Food intake (food frequency questionnaire and dietary recall)	X	X	X	X		
Familiarity with healthy food products	X	X	X	X		
Food preferences	X	X	X	X		
Household information						
Parental weight and height				X		
Parenting styles						X
Parenting practices regarding nutrition and physical activity (CSPQ)				X		
Parental well-being (SWLS)				X		
Parental HR-QoL (EQ-5D)				X		
Parental labour participation				X		
SES (including deprivation, income, and education)				X		
Parental leave and absence due to illness of child				X		
Teacher-related variables						
Teacher’s practices regarding nutrition and physical activity					X	
Teacher’s height and weight					X	
School achievement of child						
Test results (CITO and other tests)					X	
School advice and secondary school track actually attended					X	
Learning disabilities					X	
School absenteeism and repeating years					X	
Process-evaluation						
Qualitative and quantitative evaluation	X	X	X	X		
New school entries					X	

*Abbreviations*: *CITO* centrale eindtoets basisonderwijs, *CSPQ* comprehensive snack parenting questionnaire, *EQ-5D-Y* EuroQol 5-Dimensions Youth version, *EQ-5D* EuroQol 5-Dimensions, *GGD* regional public health services, *PedsQL* paediatric quality of life inventory, *SDQ* strength and difficulties questionnaire, *SEQ-C* self- efficacy questionnaire for children, *SWLS* satisfaction with life survey

#### Children’s general health

Weight is measured to the nearest 0.1 kg (Weighing Scale 803, Seca, Hamburg, Germany) and height is measured to the nearest 0.1 cm (Stadiometer 213, Seca, Birmingham, United Kingdom). Children are measured with light clothing and no shoes. BMI *Z*-scores are calculated using Dutch reference values [32]. The primary outcome measure is the absolute change in BMI *Z*-score, as we aim for BMI scores closer to the national and international standards with smaller standard deviations, which will be reflected in a decrease of BMI in overweight and obese children and an increase of BMI in underweight children. Hip and waist circumferences are measured with a measuring tape to the nearest 0.1 cm, following the World Health Organization’s assessment protocol (model 201, Seca, Hamburg, Germany) [33]. Handgrip strength is measured with a calibrated Jamar hydraulic hand dynamometer (model 5030 J1, Jamar, Huthwaite, United Kingdom) to the nearest 0.5 kg [34]. All anthropometric measurements are performed twice, and a third measurement is conducted if the difference between the first two measurements exceeds a pre-set limit (weight ≥ 0.2 kg, height ≥ 0.5 cm, hip and waist circumference ≥ 1.0 cm, handgrip strength ≥ 1 kg).

Disease status since birth, hospital admissions (number and duration), healthcare visits (number), and medication use in the previous 12 months are measured by the online parental questionnaire. The wording of all disease status items are based on a questionnaire used in a previous study, tailored to the school-age population [35]. Blood pressure measured between the ages of 4 to 6, birth weight and additional information on disease status are obtained from the regular GGD measurements.

#### Children’s health-related quality of life and psychosocial functioning

The validated EuroQol 5-Dimensions Youth version questionnaire (EQ-5D-Y) and the proxy version for parents are used to measure children’s HR-QoL [36]. Child-specific HR-QoL is measured by the validated Paediatric Quality of Life Inventory (PedsQL) and parents complete the proxy version of this questionnaire [37, 38]. Psychological attributes (emotional symptoms, conduct problems, hyperactivity/inattention, peer relationship problems and prosocial behaviour) are measured by means of the Strength and Difficulties Questionnaire, a well-validated index of psychosocial functioning in children [39, 40].

Social, emotional, and academic self-efficacy is assessed in younger children using three questions from the Self-Efficacy Questionnaire for Children (SEQ-C), which is rated using a Manikin scale [41, 42]. Children in groups 7–8 complete the full 24-item version of the SEQ-C [42]. Self-confidence, social skills, self-efficacy, school well-being, future expectations, and social support are measured by the OnderwijsMonitor Limburg programme.

#### Children’s physical activity behaviour

Physical activity levels are assessed objectively using the Actigraph accelerometer (Actigraph, GT3X+, Actigraph, Pensacola, United States). The monitor is attached to the right hip with an elastic band. All children are instructed to wear the device for seven consecutive days. The device should be worn all day except during sleeping hours and activities involving water (e.g., swimming, bathing, or showering). In the same week, parents fill in a short activity diary on their child’s physical activity and swimming behaviour and exceptional circumstances (e.g., illness of the child). In each participating school, ten randomly selected children in group 5 are equipped with a second activity monitor for 1 week, the ActivPal accelerometer (VTaP, ActivPal, Glasgow, Scotland). This accelerometer is attached to the right upper leg using tape. The ActivPal accelerometer measures postural allocation more accurately than the Actigraph, enabling us to detect sedentary behaviour patterns in more detail [43].

During PE lessons, children perform a 20-m shuttle run test, better known as the Progressive Aerobic Cardiovascular Endurance Run (PACER) as a measure of their cardiorespiratory fitness [44]. Children are encouraged to continue the test until exhaustion. Children do not perform the shuttle run test while wearing the accelerometer, as the aim is to measure usual activity patterns during a regular school week. Self-reported sports club membership, active forms of transport to school, and leisure time physical activities (e.g., children’s activities in weekends: watching TV, music or theatre, playing outdoors, practicing sports etc.) are assessed in both children and parents.

#### Children’s dietary behaviour

Food intake is measured by a combination of a food frequency questionnaire and a dietary recall tool to be completed by both children and parents. The food intake questionnaire was designed for this target group based on two questionnaires developed by Van Assema et al. [45, 46]. Items of this questionnaire include fruit and vegetables, soft drinks, sports and energy drinks, and snacks. Dietary recall is used to assess the composition of breakfast and lunch. The questions about lunch intake are asked in the classroom immediately after the lunch break. Children are not pre-informed that they are going to be asked about dietary consumption, to avoid a potential effect of the questionnaires on children’s dietary patterns.

In addition, food preferences and familiarity with healthy food products are assessed. The questions mainly consist of pictures of food items, for which children can indicate whether they have ever eaten these items and whether they like them or not. These questions were developed based on an existing instrument previously used in the INPACT study [47].

#### Household characteristics

Parental BMI is assessed by self-reported height and weight of both parents/caregivers. Parental practices regarding nutrition are determined by a shortened version (nine items) of the Comprehensive Snack Parenting Questionnaire (CSPQ) [48]. Parental practices regarding physical activity are assessed by a questionnaire developed in the same style as the CSPQ. Parental well-being and HR-QOL are measured by the Satisfaction With Life Survey (SWLS) questionnaire and the EuroQol – 5-Dimensions Questionnaire (EQ-5D), respectively [49, 50].

Labour participation of parents is assessed by current employment status. Current employment status is combined with parental education level and household income to determine SES. All questions about SES have been derived from previous, large-scale Dutch studies, including those specifically addressing socio-economic inequalities in health [51–53]. In addition, parents are asked about their ethnicity and level of (material) deprivation [54]. Parental sick leave and absence from work or education because of illness of their child is also included in the online questionnaire. Labour participation is combined with parental sick leave rates to determine productivity losses from work.

#### Teacher-related variables

Teacher’s self-reported weight and height and transport forms to work are assessed by means of a written questionnaire. School practices regarding nutrition and physical activity (e.g., modelling eating healthy food products and encouraging children’s physical activity) are determined by an adapted version of the Parental Practices Instrument.

#### Children’s academic achievements

Academic performance is monitored using the *Centrale Eindtoets Basisonderwijs* (CITO), and various other tests used by the schools [http://www.cito.com/about_cito/history_of_cito]. In the Netherlands there is a long tradition of testing at the end of primary school to support the decision in which track children will continue their secondary education. Since 2015, participation in these tests is obligatory by law. All schools participating in the study are currently using the standard version, although schools can opt for a few alternative tests. The test measures language, maths and world orientation. In addition to this end of primary school test, many schools use a wide range of tests throughout the children’s school careers. This also includes tests on mathematics (taken twice a year) and various aspects of language such as decoding skills, spelling, vocabulary, and reading comprehension. With the exception of the two partial intervention schools, the schools in this study are using a wide range of such tests, which are – using the data from OnderwijsMonitor Limburg – also comparable to those used in other schools in the region.

The secondary school advice provided by the primary school, the actual level of secondary school opted for (Dutch secondary education is hierarchically ordered), the school absenteeism and repeating years are assessed for all participating children via the schools.

#### Process evaluation

General parental satisfaction with their children’s school (including safety, communication, quality of education, challenges to children, and professionalism of teachers) are assessed using a school satisfaction questionnaire, which is regularly used in the Dutch school system. Implementation of the intervention is evaluated by qualitative outcome measures such as interviews with parents and children, and classroom observations.

In the Netherlands, parents are free to choose which school their child attends. The introduction of the intervention might therefore influence their choice of school and thus change the composition of the sample. Distance is an important determinant of school choice [26]. A simulation using the model of Borghans et al. shows that the effect on the enrolment is expected to be rather small [26]. The data provided by the OnderwijsMonitor Limburg allows us to investigate the actual choice patterns and outcomes for the schools in the region. In addition, head teachers are asked if the presence of the intervention influenced the new school entries.

#### Evaluation of legal aspects

The research questions on legal aspects will be addressed by a thorough scientific literature study and examination of policy and legislation instruments and case-law on the scope of children’s right to health. The literature study will focus on the right to health, evaluation of the scope of this right, and the right to health of children in particular. In addition, literature and case-law research will be conducted to explore the existing conflicting interests of parties involved in the realization of children’s right to health, particularly in healthy school settings. Interviews will be held to determine the interests of the different parties involved in the healthy school setting. In addition, a comparison will be made with the legal situation in Scotland, in view of the embedding of the healthy school concept in the Scottish legal (educational) system. Conflicts of interest regarding the realization of children’s rights to health and education in the healthy school setting will be explored and resolved using a legal theoretical framework. In addition, the Scottish model will be used to identify and resolve the conflicts of interest.

### Statistics

#### Sample size calculation for primary research question

The primary sample size calculation is based on the primary outcome, i.e. detecting a difference in mean absolute BMI *Z*-scores between participants in the two full-intervention schools and participants in the four control schools, after 4 years of intervention. All assumptions are shown in Table 4.

**Table 4:** Assumptions of the sample size calculation

Sample size calculation assumption
100 participants per school
2 full intervention, 2 partial intervention and 4 control schools
Participants of cohorts F, G, H, and I will be included (Table 1)
A significance level (alpha) of 0.05
A power of 80 %
Independent-samples *t*-test on difference in absolute BMI *Z*-scores
An intraclass correlation coefficient of 0.01, as based on Amorim et al. [64]
Unequal cluster sizes with a relative efficiency of 90 % [65]
A dropout rate of 20 % (including both study drop-out and natural drop-out such as migration)
An average absolute BMI *Z* of 0.76 and an SD of 0.60 in the population (these values were calculated over all children aged between 4 and 11 living in Parkstad region who visited the Youth Health Services in 2013).

With these assumptions we should be able to detect a difference in mean absolute BMI *Z*-score of 0.24 between the full-intervention and control groups, which correspondents to a medium standardized effect size (Cohen’s *d* of 0.41). Detecting this difference is feasible in this population, as a school-based longitudinal study with comparable intervention components found a mean difference between the intervention and control groups of −0.26 (CI 95 %: −0.32, −0.21) BMI *Z*-score [55].

#### Sample size calculation for secondary research question

To detect differences between the two full-intervention schools and the two partial-intervention schools, a second sample size calculation was performed. The assumptions used are shown in Table 4. With these assumptions we should be able to detect a difference in mean absolute BMI *Z*-score of 0.28 between full intervention and partial intervention, corresponding to a medium standardized effect size (Cohen’s d = 0.47). Based on the meta-analysis by Waters et al., it is likely that changes between full intervention and partial intervention of up to 0.28 can be found [56].

#### Data analysis for primary and secondary research questions

Linear mixed model analysis techniques will be used to assess the longitudinal effect of the intervention on the primary outcome measure of absolute BMI *Z*-scores. This technique corrects for correlations within individuals within groups, which occurs in this type of repeated-measures research design. Another advantage of this technique is that it naturally handles missing values in the outcome (likelihood-based method), as long as data are missing at random. Since measurements are repeated within participants, who are nested within schools, we will use a three-level model with schools as the third level, participants as the second level and measurements as the first level. The fixed part of the model consists of group (intervention versus control), time (time points at which the measurements are taken) and the interaction term group*time. Baseline variables that are related to missing data or outcomes will also be included to obtain unbiased results and/or to gain precision. As for the random part of the model, a random school and participant effect will be included in addition to the repeated measurements. For the repeated measurements, the covariance structure will be chosen based on Akaike’s Information Criterion. All participants with at least one outcome measurement will be included in the analysis.

#### Data analysis for tertiary research questions

The tertiary research questions will be answered using different statistical techniques. The longitudinal effects on numerical quantitative outcome variables will be assessed using the same linear mixed models used for the primary outcome. Categorical outcome variables will be assessed using a logistic mixed-model analysis technique, where the model is similar to that described for the primary study parameter.

Both short-term (2 years) and lifetime cost-effectiveness analyses will be conducted. School-aged children and their caregivers form the target population of the analyses. Cost-effectiveness ratios will be estimated indicating the incremental costs (investments) per unit of incremental benefit. Costs and effects will be assessed from a societal perspective, including healthcare, patient and family costs. Cost-effectiveness ratios will include the incremental costs per quality-adjusted life year gained. The number of quality-adjusted life years of primary school children and their caregivers will be calculated using self-reported HR-QoL (EQ5D) and valuations obtained from the general public [57]. The short-term cost-effectiveness analysis will use the effects from baseline to follow-up at year two. Lifetime cost-effectiveness will be studied by means of a two-step approach. First, childhood outcomes will be extrapolated to young adulthood based on existing tracking studies and epidemiologic models. Subsequently, decision-analytical modelling will be used to simulate the associated consequences in terms of duration and quality of life and societal costs over the remaining lifetime.

Qualitative data such as interviews and focus groups will be recorded, transcribed and coded using Nvivo software. Coding will be performed by two independent researchers. Peer consultation between researchers will take place frequently. Major themes and discrepancies will be discussed with a third senior researcher. All researchers involved in data collection and analyses will keep a self-reflective diary to evaluate their own subjective views on the interpretation of the data.

## Discussion

This quasi-experimental study is expected to show whether an innovative school-based health intervention effectively contributes to a wide range of outcome parameters. The main outcome measures on which effects are expected are the children’s BMI distributions (primary outcome measure), lifestyle behaviours, academic achievement, child well-being, socio-economic differences, legal implications, and societal costs.

A strength of the study design is that it focuses on outcome parameters from a wide range of scientific disciplines (i.e., medical, behavioural, psychological, educational, legal, and economic). All elements necessary for political decision-making, such as effectiveness, feasibility, cost-effectiveness and legitimacy, are included in this study design. This enables us to extrapolate our research findings to a multidisciplinary level, which can contribute not only to new scientific insights, but also to the societal and political debate about the current educational system.

The quasi-experimental design of this study has several benefits. We were able to enrol schools in the interventions on the basis of motivation, which reflects the real-life situation of public health interventions. This will facilitate implementation and continuation of the interventions in the long term [58]. A quasi-experimental design also has its limitations, however. The lack of randomization can result in selection bias at both the school and individual levels. For example, it is known that participants with a low SES are usually underrepresented in scientific studies [59]. To assess the degree of self-selection at both school and individual level, we will compare features of our study population with information from several (public) Dutch databases. Features will include academic achievement, obesity rates, health behaviours, and SES. Potential sources of selection will be included as confounders in our analyses if necessary, to reduce selection bias in the estimates. The process evaluation will also be used to assess potential forms of bias.

The duration of this study (4 years) enables us to measure long-term effects. However, a known disadvantage of such a longitudinal study over an extended period of time is the risk of a high dropout rate. We will try to motivate children and parents each year with small incentives to promote a high response rate. Still, a dropout rate of 20 % has been taken into account in the power calculation.

A difficulty of the current study design is the young age of the study population (4 to 12 years). In this age category, emotional and social parameters are difficult to measure, and validated questionnaires are often lacking. Another measurement concern is the use of self-reported outcome measures, such as dietary recall. We will minimize these potential forms of measurement error by comparing differences over time using multilevel analysis techniques. Finally, it would have been interesting to measure biomarkers of lifestyle-related changes such as triglycerides and blood glucose. Yet, we purposely chose to exclude invasive outcome measures to maximize the response rate.

The multidisciplinary character of this scientific evaluation should facilitate adoption and implementation of the intervention in other Dutch schools. Hence, this unique study can be of great societal and scientific importance. It can affect cultural and legal aspects of our society such as values and legal responsibilities regarding parenting, educational laws, the exemplary function of the school, and labour input by parents or caregivers.

## Abbreviations

BMI, body mass index; CITO, Centrale Eindtoets Basisonderwijs (Test developed by Dutch Central Institute for Test Development); CSPQ, comprehensive snack parenting questionnaire; EQ-5D, EuroQol 5 dimensions; EQ-5D-Y, EuroQol 5 Dimensions Youth version; HR-QoL, health-related quality of life; GGD: Geneeskundige Gezondheidsdienst (regional Public Health Services); MEC, medical ethics committee; PE, physical education; PedsQL, paediatric quality of life inventory; SDQ, Strength and Difficulties Questionnaire; SEQ-C, self-efficacy questionnaire for Children; SES, socio-economic status; SWLS: satisfaction with life survey
