# Erratum to: The Healthy Primary School of the Future: study protocol of a quasi-experimental study

**DOI:** 10.1186/s12889-017-4230-y

**Published:** 2017-04-11

**Authors:** M. Willeboordse, M. W. Jansen, S. N. van den Heijkant, A. Simons, B. Winkens, R. H. M. de Groot, N. Bartelink, S. P. Kremers, P. van Assema, H. H. Savelberg, E. de Neubourg, L. Borghans, T. Schils, K. M. Coppens, R. Dietvorst, R. ten Hoopen, F. Coomans, S. Klosse, M. H. J. Conjaerts, M. Oosterhoff, M. A. Joore, I. Ferreira, P. Muris, H. Bosma, H. L. Toppenberg, C. P. van Schayck

**Affiliations:** 1 Department of Family Medicine, School of Public Health and Primary Care (CAPHRI), Maastricht University, Maastricht, The Netherlands; 2 Academic Collaborative Centre for Public Health Limburg, Public Health Services, Geleen, The Netherlands; 3 Department of Health Services Research, CAPHRI, Maastricht University, Maastricht, The Netherlands; 4 MOVARE Educational board, Kerkrade, The Netherlands; 5 Department of Methodology and Statistics, CAPHRI, Maastricht University, Maastricht, The Netherlands; 6 Welten Institute - Research Centre for Learning, Teaching and Technology, Open University of the Netherlands, Heerlen, The Netherlands; 7 School of Nutrition and Translational Research in Metabolism (NUTRIM), Maastricht University, Maastricht, The Netherlands; 8 Department of Health Promotion, CAPHRI, Maastricht University, Maastricht, The Netherlands; 9 Department of Health Promotion, NUTRIM, Maastricht University, Maastricht, The Netherlands; 10 Department of Human Movement Sciences, NUTRIM, Maastricht University, Maastricht, The Netherlands; 11 School of Business and Economics, Maastricht University, Maastricht, The Netherlands; 12 Department of Law, Maastricht University, Maastricht, The Netherlands; 13 Academic Hospital Maastricht, Treatment and Care Unit, Maastricht, The Netherlands; 14 Department of Clinical Epidemiology and Medical Technology Assessment, CAPHRI, Maastricht University, Maastricht, The Netherlands; 15 School of Public Health, The University of Queensland, Herston, Brisbane, Australia; 16 Department of Clinical Psychological Science, Faculty of Psychology and Neuroscience, Maastricht, The Netherlands; 17 Department of Social Medicine, CAPHRI, Maastricht University, Maastricht, The Netherlands

## Erratum

Following publication of this article [1], it has come to our attention that some of the terminology used in the article could be difficult to interpret. In table 1, the description of the measurements should begin with t0. This would mean that t1 becomes t0, t2 becomes t1, and so forth. Furthermore, where schoolchildren are referred to in groups 1–8, groups 1 and 2 refer to Kindergarten children aged 4–6 years, and groups 3–8 correspond to children in Grades 1–6 aged 6-12 years. The correct abbreviation for 'The Healthy primary School of the Future' is HPSF.
